# Clogging sensitivity of flow distributors designed for radially elongated hexagonal pillar array columns: a computational modelling

**DOI:** 10.1038/s41598-021-84178-w

**Published:** 2021-03-02

**Authors:** Farideh Haghighi, Zahra Talebpour, Amir Sanati-Nezhad

**Affiliations:** 1 Department of Chemistry, Faculty of Physics and Chemistry, Alzahra University, Vanak, Tehran Iran; 2 BioMEMS and Bioinspired Microfluidic Laboratory, Department of Mechanical and Manufacturing Engineering, Centre for Bioengineering Research and Education (CBRE), Biomedical Engineering Program, University of Calgary, Mechanical Engineering Building, MEB214, 2500 University Dr., N.W., Calgary, AB T2N 1N4 Canada

**Keywords:** Analytical chemistry, Lab-on-a-chip, Chemical engineering, Mechanical engineering

## Abstract

Flow distributor located at the beginning of the micromachined pillar array column (PAC) has significant roles in uniform distribution of flow through separation channels and thus separation efficiency. Chip manufacturing artifacts, contaminated solvents, and complex matrix of samples may contribute to clogging of the microfabricated channels, affect the distribution of the sample, and alter the performance of both natural and engineered systems. An even fluid distribution must be achieved cross-sectionally through careful design of flow distributors and minimizing the sensitivity to clogging in order to reach satisfactory separation efficiency. Given the difficulty to investigate experimentally a high number of clogging conditions and geometries, this work exploits a computational fluid dynamic model to investigate the effect of various design parameters on the performance of flow distributors in equally spreading the flow along the separation channels in the presence of different degrees of clogging. An array of radially elongated hexagonal pillars was selected for the separation channel (column). The design parameters include channel width, distributor width, aspect ratio of the pillars, and number of contact zone rows. The performance of known flow distributors, including bifurcating (BF), radially interconnected (RI), and recently introduced mixed-mode (MM_I_) in addition to two new distributors designed in this work (MM_II_ and MM_III_) were investigated in terms of mean elution time, volumetric variance, asymmetry factors, and pressure drop between the inlet and the monitor line for each design. The results show that except for pressure drop, the channel width and aspect ratio of the pillars has no significant influence on flow distribution pattern in non-clogged distributors. However, the behavior of flow distributors in response to clogging was found to be dependent on width of the channels. Also increasing the distributor width and number of contact zone rows after the first splitting stage showed no improvement in the ability to alleviate the clogging. MM_I_ distributor with the channel width of 3 µm, aspect ratio of the pillars equal to 20, number of exits of 8, and number of contact zones of 3 exhibited the highest stability and minimum sensitivity to different degrees of clogging.

## Introduction

In majority of microfluidic devices used for separation of molecules, such as capillary electrochromatography^1^, hydrodynamic chromatography^2^, or high performance liquid chromatography^3^, the sample and carrier fluid are driven through microfabricated channels with the largest possible width to increase total volume of sample flow within the device. Equally distribution of the sample and carrier fluid’s band through such network of channels remains a problem. In microfabricated liquid chromatography channels, stationary phase can be introduced into four different column types: open-tubular, particle-packed, monolithic, and pillar array columns (PAC)^4,5^. In each type of columns, different geometrical parameters could affect the separation and performance of the system. Several experimental and numerical studies have investigated the impact of conduit geometry and particle aspect ratio on the packing and separation efficiency of particle packed columns^6–10^.

After Knox’s suggestion to employ a uniform design of structures for improving the separation efficiency of chromatographic systems^11^, Regnier^12^ introduced a highly ordered micromachined pillar array column as a separation channel consisting of regular arrays of cubic structures with identical sizes. This was the beginning of intensive studies on the PACs to investigate the effect of pillars’ shape and size on separation efficiency. Three major types of pillar shapes (cylindrical, hexagonal, and diamond-like) have been hitherto arranged with different side-walls, interpillar distances, or radial and axial pillar lengths to create effective PACs^13^. Subsequently, the pillar arrays are microfabricated on the substrate, therefore the size, shape, and position of each individual pillar can be easily manipulated^14,15^. Radially elongated hexagonal pillars with high aspect ratio ($AR={w}_{p}/{l}_{p}$) have appeared to outperform the other pillar shapes^16,17^, where w_p_ and l_p_ denoting the width and length of the pillars, respectively. The axial molecular diffusion reduces in radially elongated pillars^18^. Additionaly, it is well established that the sidewall effect is entirely absent in designs with AR > 15 owing to high tortuosity. Despite the fact that the pressure drop increases with the increase in tortuosity^18^, experimental and theoretical studies have shown that the radially elongated diamond-like pillars can produce identical separation performance as an open-tubular column^19,20^.

Given that the entrance channel of PACs is narrower than the separation channel width, mobile phase needs to distribute evenly across the cross-section of the separation channel. Therefore, flow distributors have been devised at the entrance of the separation channel and exit of the flow collector to equivalently spread the flow along the separation channel and recollect the sample into a narrow exit channel, respectively. Various factors may contribute to clogging or fouling of microfabricated channels, including precipitated buffer, insoluble sample in the mobile phase, a component or high-molecular weight constituents of the sample that are strongly adsorbed to the column, strongly retained contaminants, deposition and assembly of debris in the mobile phase, entrapment of air bubbles as well as flaws introduced to the structure during chip manufacturing^21^. In every case of clogging either from precipitated salts or entrapment of air bubbles, the spatiotemporal clogging induces an increase in flow resistance and a drop in velocity proportional to the clogging’s cross-section. The channel clogging may affect distribution of the sample within the distributor and alter the performance of both natural and engineered systems^22^. Therefore, an even fluid distribution must be achieved cross-sectionally through careful design of flow distributors in order to reach a satisfactory separation efficiency through minimizing the sensitivity to clogging. Different designs of flow distributors with various shapes of pillars and geometries of the separation channel have been introduced and examined in last 10 years. These studies reported the significant impact of the geometry of flow distributors on shape of the eluting tracer^14^.

Among all reported flow distributors, bifurcating (BF) and radially diverging interconnected (RI) distributors are the known flow distributors that have been integrated with an array of high aspect ratio radially elongated separation channels. BF distributor consists of a network of bifurcating but non-interconnected channels, resulted in identical flow paths upon flow bifurcation. In contrast, RI distributor consists of high AR hexagonal pillar arrays with the flow distributing from the narrow supply to the wide PAC with a divergence angle less than 180°, resulted in a bundle of homogeneous interconnecting channels with frequent mixing nodes^23^. Therefore, in the RI architecture, the velocity at the center of the distributor is higher than the sidewalls, causing band broadening and peak distortion. Considering their less sensitivity to local clogging, RI distributors outperform their BF counterparts in the presence of clogging. If one of the channels in the RI distributor gets clogged (e.g. because of sample residues, microfabrication artifacts, etc.), the flow paths would contact again to tackle the clogging. As a result, when no clogging exists, BF outperforms because of its minimal volume (as little contribution to band broadening as possible) and identical flow paths^22,24^.

Jespers and his colleagues^22^ recently introduced a new type of flow distributor called “mixed-mode”. Mixed-mode distributor is a combination of BF and RI distributors which represents the positive features of both BF and RI distributors all in one unit. In this distributor design, a contact zone was incorporated following each bifurcating stage. Jespers’ team studied the effect of clogging on different flow distributor designs integrated with an array of flat-rectangular pillars (AR = 12) in a 2.5 μm-wide separation channel. Among all the platforms studied, the so-called ‘MM_I_’ was identified less sensitive to clogging, suggesting that increasing the number of distributor row near the inlet may contribute to less clogging sensitivity when the probability of clogging is higher^22^. However, the design in Ref^22^ presented the effect of clogging only for a certain value of AR and channels width. Given that the inevitable clogging may occur in different locations, numbers, shapes, and degrees of clogging along the distributors in diverse experimental testing conditions, experimental creation of all possible clogging scenarios in different locations and with different degrees of clogging comes with complications. There is a lack of optimal distributor design with minimal or no dependency to the location of clogging.

In this study, we employed computational fluid dynamics (CFD) to compare flow distributors in terms of their ability to distribute a small sample volume over the entire width of radially elongated hexagonal pillars array column in the absence or presence of different percentages of clogging. Five different flow distributors were examined in this study where they were interconnected to an array of radially elongated hexagonal pillars. All distributors have the same inlet and number of outlets (distinct locations where the fluid leaves the distributor), and need to handle the same linear velocity unless otherwise stated. The work simulates various channel widths, aspect ratios of pillars, distributor width, and number of contact zone rows to examine the effect of geometrical parameters of distributors and clogging on the performance of flow distributors. The performance of each flow distributor was evaluated numerically in terms of mean elution times, volumetric variances, asymmetry factors, and pressure drop between the inlet and the outlet, and was used to identify the optimal flow distributor design with minimal sensitivity to clogging.

## Methods

### Layouts of flow distributors

**Figure 1:**
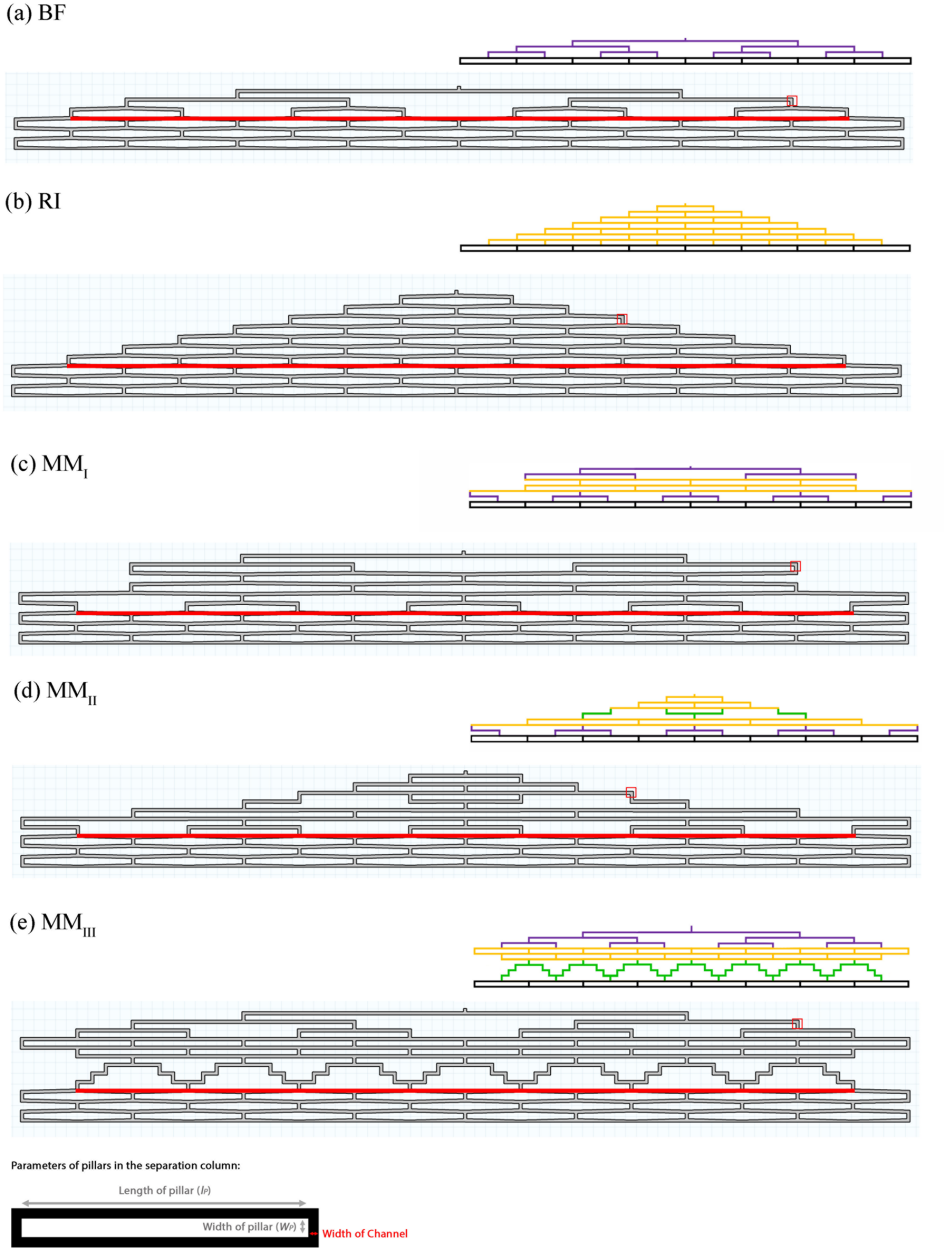
Two dimensional (2D) geometries of distributors. Lay-out for the (**a**) bifurcating (BF); (**b**) radially interconnected (RI); and (**c–e**) Mixed-mode (MM_I_, MM_II_, and MM_III_) distributors. All the flow distributors have 8 exit points. The red line in each distributor exhibits the location of the eluted band. The red box in each distributor exhibits the clogged cross-sectional area to investigate the clogging effects. The black, purple, yellow, and green channels in each distributor exhibit the separation channels, bifurcating stages, contact zones, and smaller pillars and wedges, respectively. (For interpretation of the references to colour in this figure legend, the reader is referred to the web version of this article).

The flow distributors investigated in this study and their schematics indicating each part of the flow distributors (bifurcating stages, contact zones, smaller pillars and wedges are depicted in Fig. 1. These distributors include (a) BF design in which the flow is split through n series of bifurcating channels (purple channels), creating second channels of equal length. As numerically demonstrated by Davydova and his co-workers^25^, to decrease dispersion in the BF design, an equal width was set to all bifurcation levels; (b) RI distributor with high AR hexagonal pillar arrays, including interconnecting channels with frequent mixing nodes (yellow channels); and (c–e) mixed-mode designs (MM_I_-MM_III_) containing different combination of BF, RI, and mixed-size distributors. The geometry of MM_I_ distributor design, adopted from Ref^22^, starts with bifurcating channels (purple channels), followed by contact zone rows (yellow channels), and continues with another bifurcating stage. Knowing the higher values of AR used in this research (> 15) than the Ref^22^ (AR = 15), the number of bifurcating stages for MM_I_ distributor in this study is less than Ref^22^. In contrast, the geometry of MM_II_ distributor initiates with RI design and is followed by contact zone rows in the middle of each of the splitting stage.

The geometry of MM_III_ distributor design starts with bifurcating channels, followed by rows of contact zones after the second splitting stage, and continues with a series of smaller rectangular pillars and wedges (green channels). The incorporation of contact zone rows after the splitting stages in MM designs provides more time for the fluid to redistribute after the clogged regions. The series of smaller rectangular pillars and wedges at the end of the MM_III_ distributors were designed to provide a higher chance for the entrapped species in the clogged channel to elute very slowly even below the limit of detection. Each flow distributor was connected to an array of radially elongated hexagonal pillars as the separation channel.

### Clogging study

To assess the stability of different flow distributors in response to clogging, different geometrical design parameters were considered: width of the channels throughout the entire geometry, aspect ratios of the elongated pillars (AR) in separation channel, distributor width, and number of contact zone rows consisting of interconnected channels after each of the splitting stage. Given that the smallest feature throughout the entire geometry is the distance between the two adjacent pillars, and this distance depends on the quality of etching process^26,27^, the minimum channel width was considered to be 2.5 µm. A maximum channel width of 3.5 µm was studied to consider the possible effect of dispersion in larger channels. Since the sidewall effect is no longer influential on performance of the distributor for AR > 15, a minimum AR = 15 was used throughout the study. On the other hand, a maximum AR of 25 was used in our clogging sensitivity studies to eliminate the increased pressure drop caused by an increased tortuosity.

The red box depicted in Fig. 1 was modified to reflect different clogging percentages of the 4-outlet-level channel. The chance of local clogging is higher closer to the inlet. Therefore, it induces an increased local flow resistance and a drop in velocity proportional to the clogging’s cross-section. Each distributor was first studied to figure out the effect of channel width on clogging sensitivity, then the ones with minimum sensitivity to clogging were selected to investigate the effect of aspect ratio of the pillars. Furthermore, two and three channels were modified to induce the extreme degree of clogging simultaneously, and the results were shown on the selected distributor. Also, the influence of increasing the distributor’s width and adding the number of contact zones were studied on the selected distributor.

### Numerical methods and calculations

All simulations were performed on Dell Precision Tower 7910 XCTO Base each equipped with an Intel Xeon processor E5-2630 (2.4 GHz, 16 cores) and 32 Gb, 2400 MHz ram memory, running on Linux server. Simulations were accomplished with the COMSOL Multiphysics commercial software (COMSOL 5.3) in a 2D simulation model. Each flow distributor was divided into 500,000 and 3,000,000 triangular and free quad meshes. Mesh and time independencies were examined via decreasing the mesh size and time step to half of their initial values for the BF distributor with channel width of 3 µm integrated with 100 µm wide and 5 µm thick radially elongated hexagonal pillars rows. The difference in volumetric variances was less than 2.5%.

An incompressible laminar fluid flow, governed by the Navier–Stokes equations, was computed using the stationary solver. As the boundary condition, a linear velocity was applied at the inlet of the distributor and zero pressure at the outlet of the column. All inlet velocities were chosen such that a relevant mean velocity of 0.25 mm/s was obtained in the subsequent bed. The water with a viscosity of 1 × 10^−3^ kg/ (m s) and a density of 998.2 kg/m^3^ was selected as the carrier fluid. The water was also used as the tracer with the molecular diffusion coefficient of 10^−9^ m^2^/s. The time dependent transport of diluted species governed by the Fick’s law was computed with the same mesh density as used in the velocity field calculation. The inlet plane was set to the inflow with an arbitrary concentration of the tracer. The outlet plane was set to the outflow with zero concentration. The rest of the boundaries were set to no-slip wall. Chromatograms were obtained by integrating the tracer concentration over the monitor line (the red line in each of the distributors shown in Fig. 1). The red boxes in each distributor represent the clogging zone at the 4-outlet level to study the clogging sensitivity of the distributors. The performance of each flow distributor was evaluated based on the volumetric variance (σ_v_^2^) of the tracer band exiting the distributor. To this end, peaks area (m_0_), mean elution time (m_1_), and time-based variance (m_2_, σ_t_^2^) for each simulation were calculated based on moment analysis (Eqs. (1–3))^28^.

1 $$Peak\, area \left({m}_{0}\right)={\int }_{{t}_{1}}^{{t}_{2}}C\left(t\right)dt$$

2 $$Mean\, elution\, time ({m}_{1})=\frac{{\int }_{{t}_{1}}^{{t}_{2}}tC\left(t\right)dt}{{m}_{0}}$$

3 $$Time-based\, variance ({m}_{0},{\sigma }_{t}^{2})=\frac{{\int }_{{t}_{1}}^{{t}_{2}}{(t-{m}_{1})}^{2}C\left(t\right)dt}{{m}_{0}}$$

wherein C(t), t_1_, and t_2_ refer to mass fraction of the tracer as a function of time, peak start time, and peak end times, respectively^28^. Thereafter, σ_v_^2^ is determined with Eq. (4).^22^

4 $${\sigma }_{v}^{2}={\sigma }_{t}^{2}\times {F}^{2}$$

where F is flow rate. Peak shape measurements (i.e. asymmetry factors, AsF and TF) was calculated based on the ref^28^.

## Results

### Calculating the performance of non-clogged flow distributors

**Figure 2:**
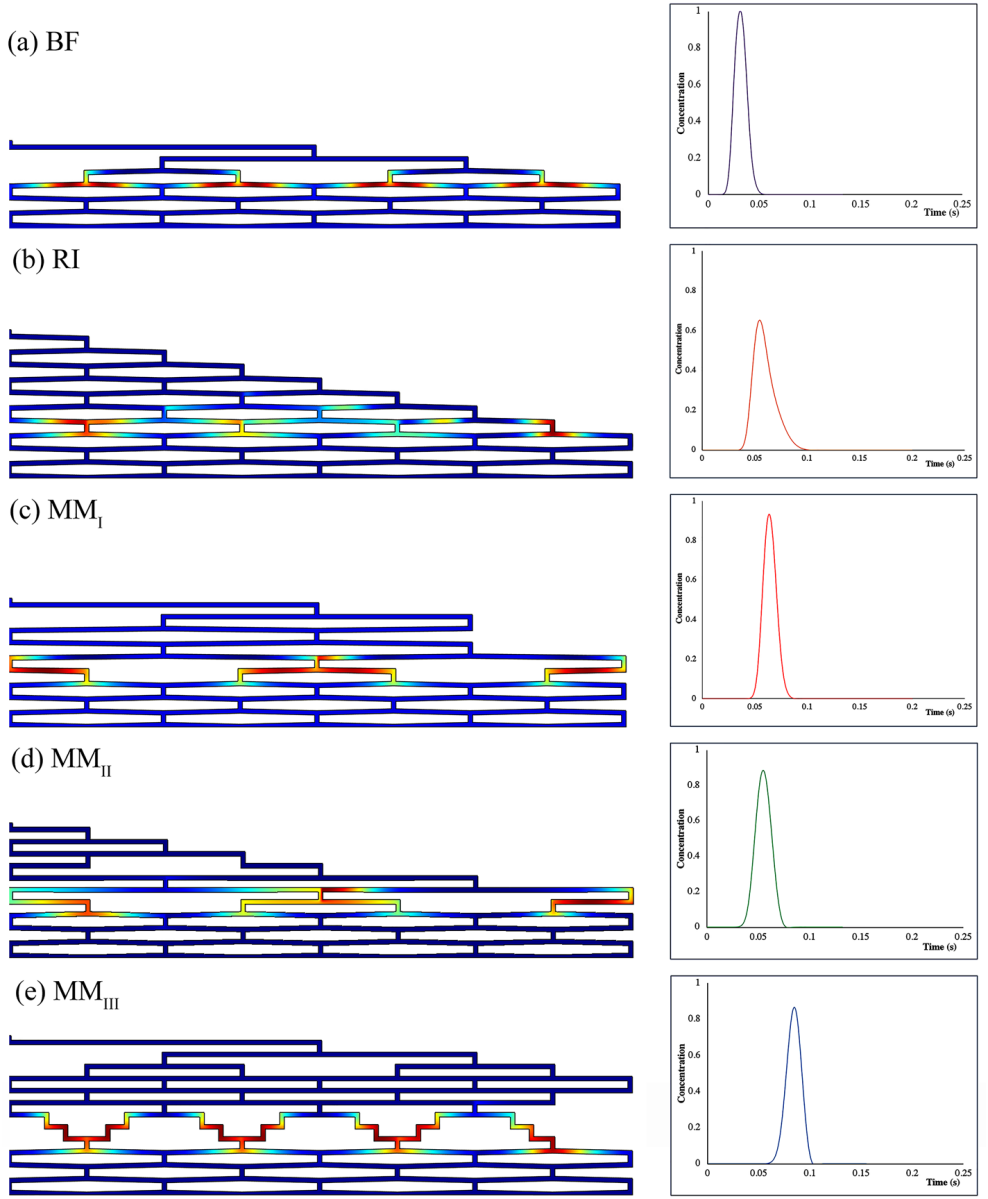
View of the band distribution migrating through the distributor prior to the moment of elution in the absence of clogging (left). The corresponding chromatograms recorded at the monitor line (right) (channel width = 3 µm, AR = 20, and flow rate = 1.32 µL/min). Color scales are linear with concentration of the tracer (red = maximum, blue = 0).

The performance of each flow distributor was first assessed in non-clogged conditions. Figure 2 depicts the concentration profile of eluting band in each flow distributor (channel width = 3 µm, AR = 20) where the tracer band reaches the monitor line (red line) along with its corresponding chromatogram.

Numerical values regarding the eluting peaks including mean elution time, volumetric variance, asymmetry factors as well as pressure drop between the inlet and the monitor line were calculated from computational simulation for each distributor and summarized in Table 1.

**Table 1:** Numerical measures comparing different flow distributors in the absence of clogging at a flow rate of 1.32 μL/min. (^a^Aspect ratio, ^b^Mean elution time, ^c^Volumetric variance, ^d^Asymmetry factor, ^e^Tailing factor, ^f^Pressure drop).

Flow distributor	Channel width (µm)	AR^a^	m_1_ (s)^b^	${\upsigma }_{\mathrm{v}}^{2}$(nL^2^)^c^	AsF (/)^d^	TF (/)^e^	Δp (bar)^f^
BF	2.5	20	0.032	0.046	1.06	1.06	0.21
	3	20	0.032	0.044	1.14	1.12	0.14
	3.5	20	0.032	0.046	1.08	1.07	0.12
RI	2.5	20	0.058	0.11	1.56	1.28	0.13
	3	20	0.059	0.15	2.31	1.69	0.095
	3.5	20	0.058	0.11	1.51	1.25	0.075
MM_I_	2.5	20	0.064	0.055	1.00	0.99	0.26
	3	15	0.064	0.054	0.99	0.99	0.12
	3	20	0.064	0.053	1.06	1.09	0.20
	3	25	0.063	0.051	0.99	0.99	0.30
	3.5	20	0.064	0.058	1.06	1.03	0.15
MM_II_	2.5	20	0.055	0.071	1.02	1.00	0.17
	3	20	0.055	0.072	1.01	1.00	0.12
	3.5	20	0.055	0.074	1.02	1.02	0.10
MM_III_	2.5	20	0.084	0.064	0.95	0.95	0.23
	3	15	0.084	0.072	0.98	0.98	0.11
	3	20	0.083	0.065	1.02	1.00	0.19
	3	25	0.082	0.062	0.95	0.95	0.25
	3.5	20	0.086	0.068	0.96	0.96	0.13

The results show that increasing the number of contact zone rows in distributors increases the total volume capacity of the system and therefore rises the elution time (changed from 0.032 s in BF to 0.084 in MM_III_). Because the dispersion is strongly governed by the volume of distributors, the increase in this volume leads to a peak broadening and distortion. Based on the results of the corresponding chromatograms (Fig. 2), BF design creates the narrowest peak (σ_v_^2^ = 0.044 nL^2^, channel width = 3 µm and AR = 20) with a relatively perfect symmetry $\cong$ 1.1 owing to its small volume, while the results of the RI distributor is a widest peak (σ_v_^2^ = 0.15 nL^2^, channel width = 3 µm and AR = 20) with asymmetry response, tailing factor higher than 1.5, and minimal pressure drops (0.075–0.13 bar) due to its interconnected structure.

**Figure 3:**
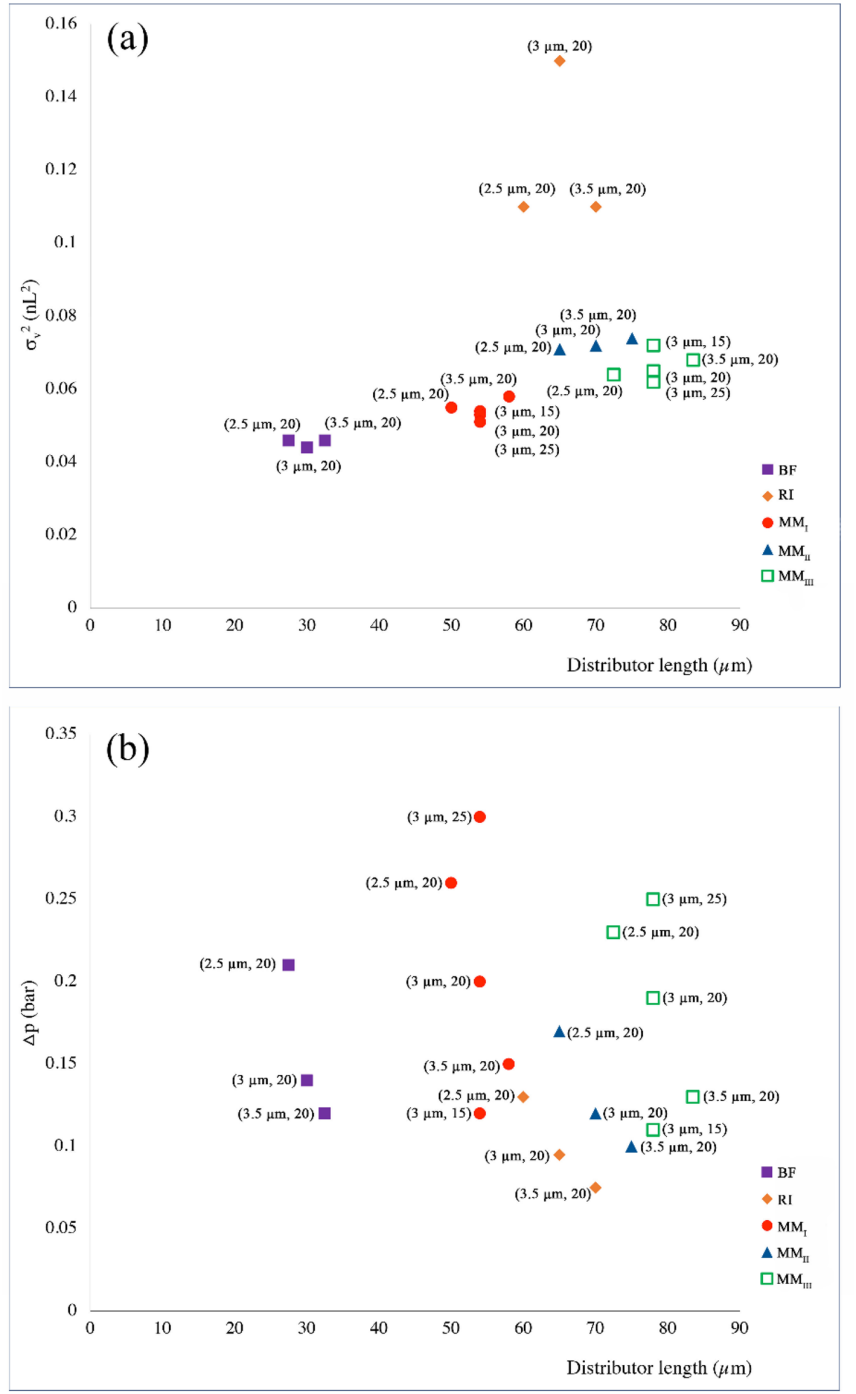
Dependence of (**a**) the peak volumetric variance and (**b**) pressure drop between inlet and monitor line to the length of the distributors.

Figure 3 represents the dependency of volumetric variance of the eluted peak and pressure drop over the distributor to the distributor length. As expected, BF distributor generated the minimum σ_v_^2^ values due to their minimal volume and length, while RI contributed to the largest σ_v_^2^ values. In each set of designs, increasing the length of the distributor increases σ_v_^2^ value slightly except for RI distributor (Fig. 3(a)). For instance, from MM_I_ with the smallest channel width of 2.5 µm to MM_I_ with the largest channel width of 3.5 µm, σ_v_^2^ value increased from 0.055 to 0.058 nL^2^.

These values vary for MM_II_ and MM_III_ from 0.071 to 0.074 nL^2^ and 0.064 to 0.068 nL^2^, respectively.

On the other hand, pressure drop over the length of each set of distributor designs decreased by increasing either the channel width or tortuosity (Fig. 3. (b)). The largest Δp values of 0.26 and 0.23 bar as a result of narrower channel width, and Δp-values of 0.30 and 0.25 bar due to increase in tortuosity were observed in MM_I_ and MM_III_ distributor designs, respectively. This phenomenon is due to the presence of rows of radially interconnected pillars with high value of AR in their flow distributor structure which increases the fluid path length and generates an extra pressure drop.

Among different MM flow distributors in this study, the peaks eluting from MM_I_ and MM_III_ have longer mean elution times (Table 1) due to the presence of more than one fluid contact zone after the flow splitting stages (Fig. 1). On the other hand, among MM-type distributors, the peak leaving the MM_II_ distributor show the highest volumetric variance (σ_v_^2^ = 0.074 nL^2^, channel width = 3 µm and AR = 20) which may be attributed to the RI structure at the entrance. All the MM-type distributors produce a completely symmetric peak with $\mathrm{AsF}\cong 1$.

Overall, among all the non-clogged distributors, RI produced the widest peaks, MM_I_ showed the maximum pressure drop, MM_III_ generated the largest mean elution time, and MM_II_ showed an intermediate response. Therefore, BF distributor outperforms in the absence of clogging with the lowest elution time and pressure drop, and perfect symmetry.

### Effect of channel width on clogging sensitivity of distributors

**Figure 4:**
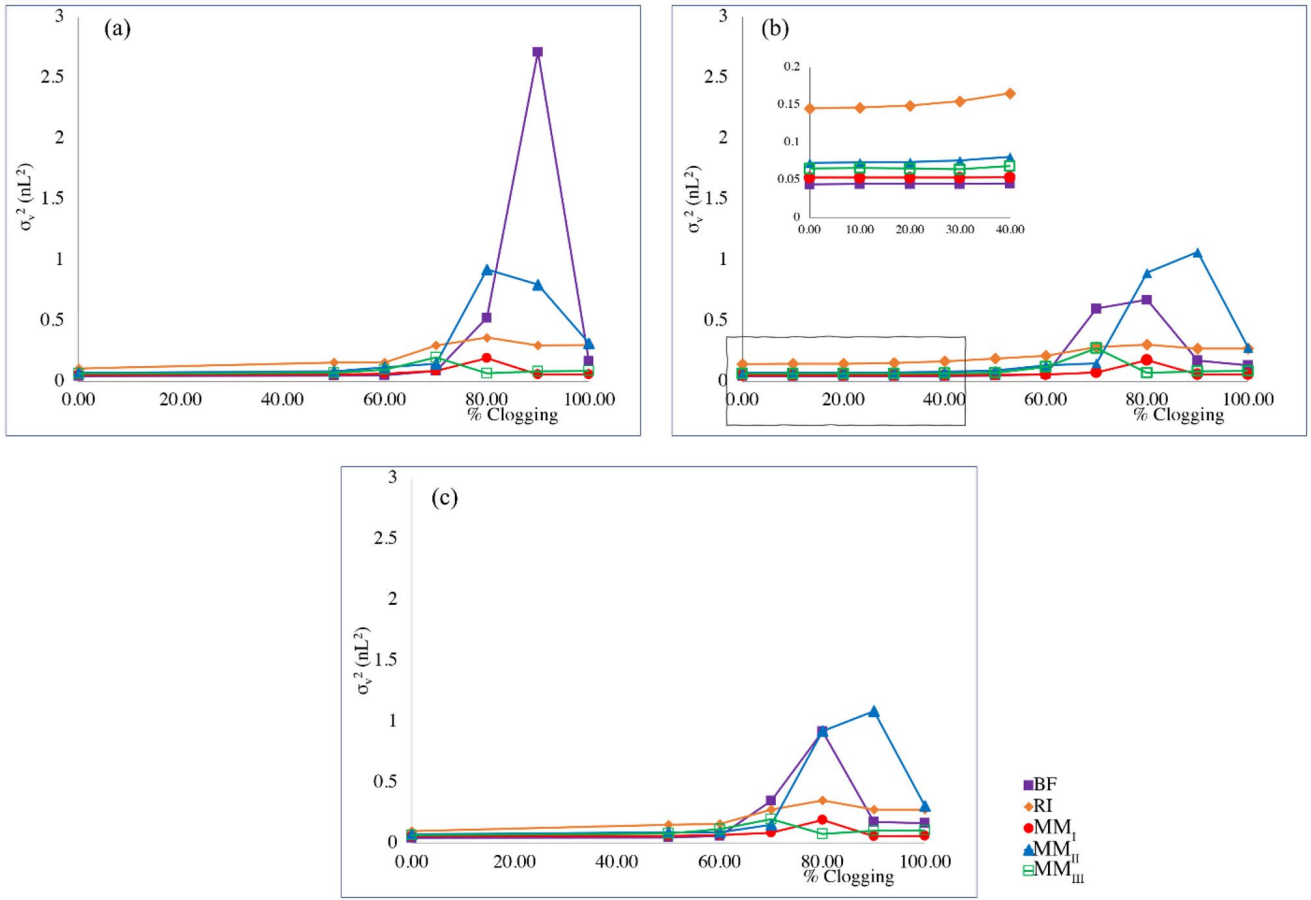
Volumetric variances of the eluted tracer recorded at the monitor line as a function of the degree of clogging in the red boxes indicated in Fig. 1 at different channel widths (**a**) 2.5, (**b**) 3, and (**c**) 3.5 µm. AR = 20. Flow rate = 1.32 µL/min. For interpretation of the references to colour in this figure legend, the reader is referred to the web version of this article.

**Figure 5:**
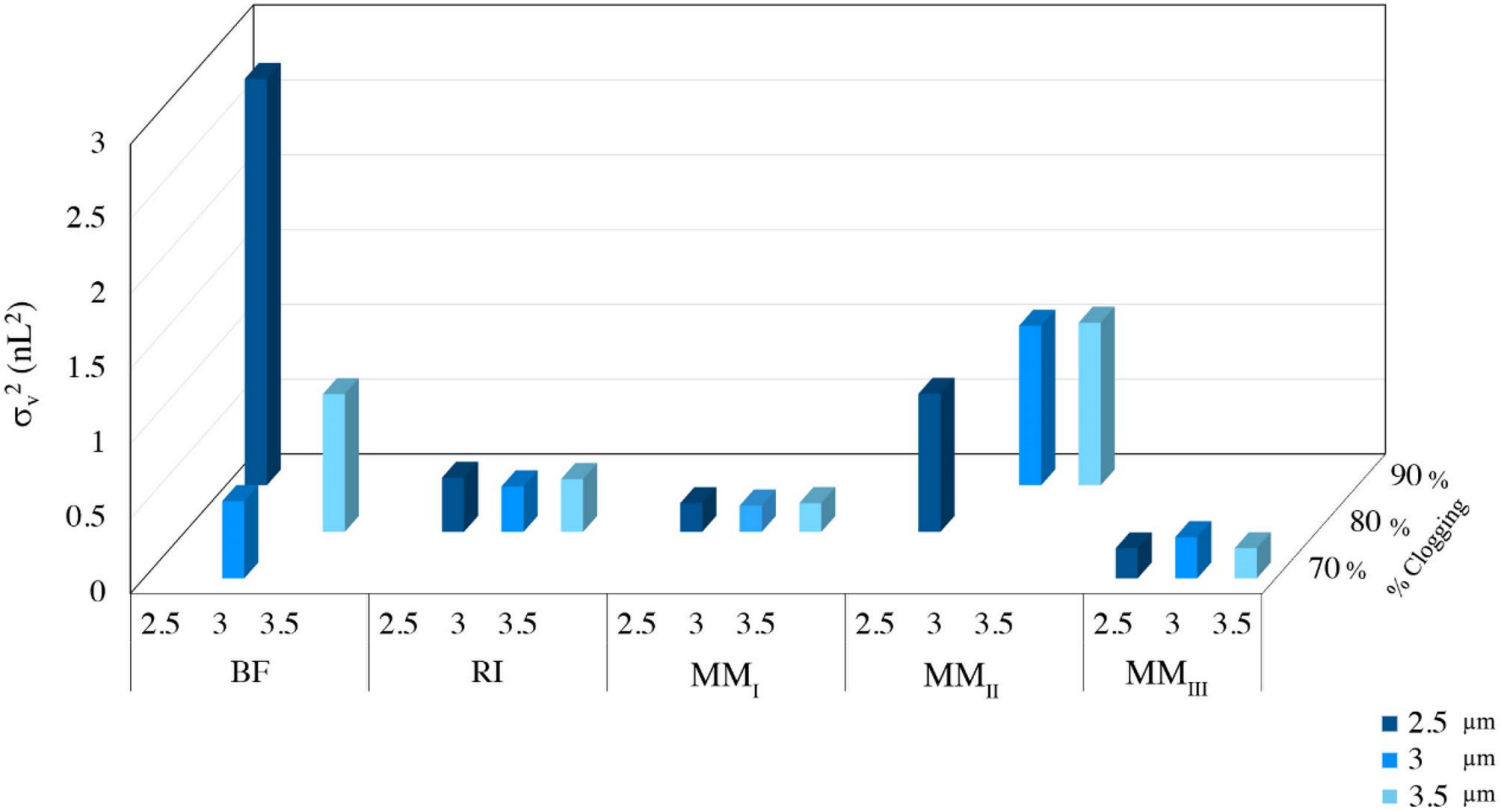
The widest eluted peaks, undesirable outcomes, for each flow distributor at the relevant degrees of clogging.

The results of clogging sensitivity for different flow distributors tested under different channel widths (2.5, 3, 3.5 µm) are shown in Fig. 4. Each distributor was fed into a separation column with a channel width equal to that of the distributor, where the AR of the elongated pillars in the separation column was fixed to 20. As explained in Methods, the percentage of clogging in the red box (represented in Fig. 1) was changed in 10 incremental steps to reach the maximum 100% clogging with the σ_v_^2^-value for each case being calculated. The results show that there are no significant changes in the σ_v_^2^ values at the first four clogging degrees (10, 20, 30, and 40%) for the channel width of 3 µm (Fig. 4 (b)), therefore these clogging steps were eliminated for the rest of the study. Figure 4 depicts that flow distributors with various channel width show different behavior in the presence of clogging. For instance, BF-type distributors are more sensitive to clogging compared to the other distributor types; BF with 2.5 µm channel width shows the widest peak (σ_v_^2^ = 2.72 nL^2^) at 90% clogging. However, for 3 µm and 3.5 µm channel widths, the widest peak occurs at the 80% clogging. Therefore, the widest peak, as an undesirable outcome for each flow distributor, may occur at different degrees of clogging. These undesirable states and the relevant degree of clogging were compared among different distributors (Fig. 5). The σ_v_^2^ values of BF and MM_II_ distributors are the largest and the most affected ones under changing the channel width, with more than 12 times higher σ_v_^2^ value than the values in the absence of clogging. However, the eluted peaks from RI-, MM_I_-, and MM_III_-type distributors are approximately 3 or 4 times wider than the eluted peaks in the absence of clogging. Also, the minimum fluctuations in σ_v_^2^ values occurs as a function of clogging percentages in RI, MM_I_, and MM_III_ distributors (Fig. 4), which shows their ability in compensating the clogging.

The numerical measures for each undesirable state of the eluted peak and the relevant degree of clogging are summarized in Table 2. Numerical measures regarding the eluted peaks produced by the non-clogged flow distributors are compared to the widest eluted peaks, undesirable states, at the relevant degree of clogging with the data summarized in Tables 1 and 2. The results show that for channel clogging exceeding 70%, the mean elution time did not increase significantly except for the BF distributor. This is where pressure drop increased approximately 0.02 bar in each flow distributor in the presence of clogging except for the MM_III_ distributor, showing negligible change in Δp after clogging. The σ_v_^2^ values for all flow distributors with undesirable eluted band becomes markedly higher at the relevant degree of clogging.

**Table 2:** Numerical measures for each undesirable state of eluted peak and its relevant clogging percentage of the 4-outlet-level channel for each set of distributors with different channel widths. The aspect ratio of pillars is set to 20. The flow rate is set to 1.32 μL/min. (^a^Mean elution time, ^b^Volumetric variance, ^c^Pressure drop).

Flow distributor	Channel width (µm)	Clogging (%)	m_1_ (s)^a^	${\upsigma }_{\mathrm{v}}^{2}$(nL^2^)^b^	Δp (bar)^c^
BF	2.5	90	0.062	2.72	0.23
	3	70	0.038	0.52	0.16
	3.5	80	0.046	0.92	0.13
RI	2.5	80	0.059	0.36	0.14
	3	80	0.058	0.32	0.11
	3.5	80	0.058	0.35	0.08
MM_I_	2.5	80	0.062	0.19	0.28
	3	80	0.062	0.17	0.22
	3.5	80	0.064	0.19	0.16
MM_II_	2.5	80	0.061	0.92	0.20
	3	90	0.059	1.06	0.15
	3.5	90	0.059	1.08	0.12
MM_III_	2.5	70	0.083	0.20	0.23
	3	70	0.081	0.27	0.19
	3.5	70	0.084	0.20	0.14

**Figure 6:**
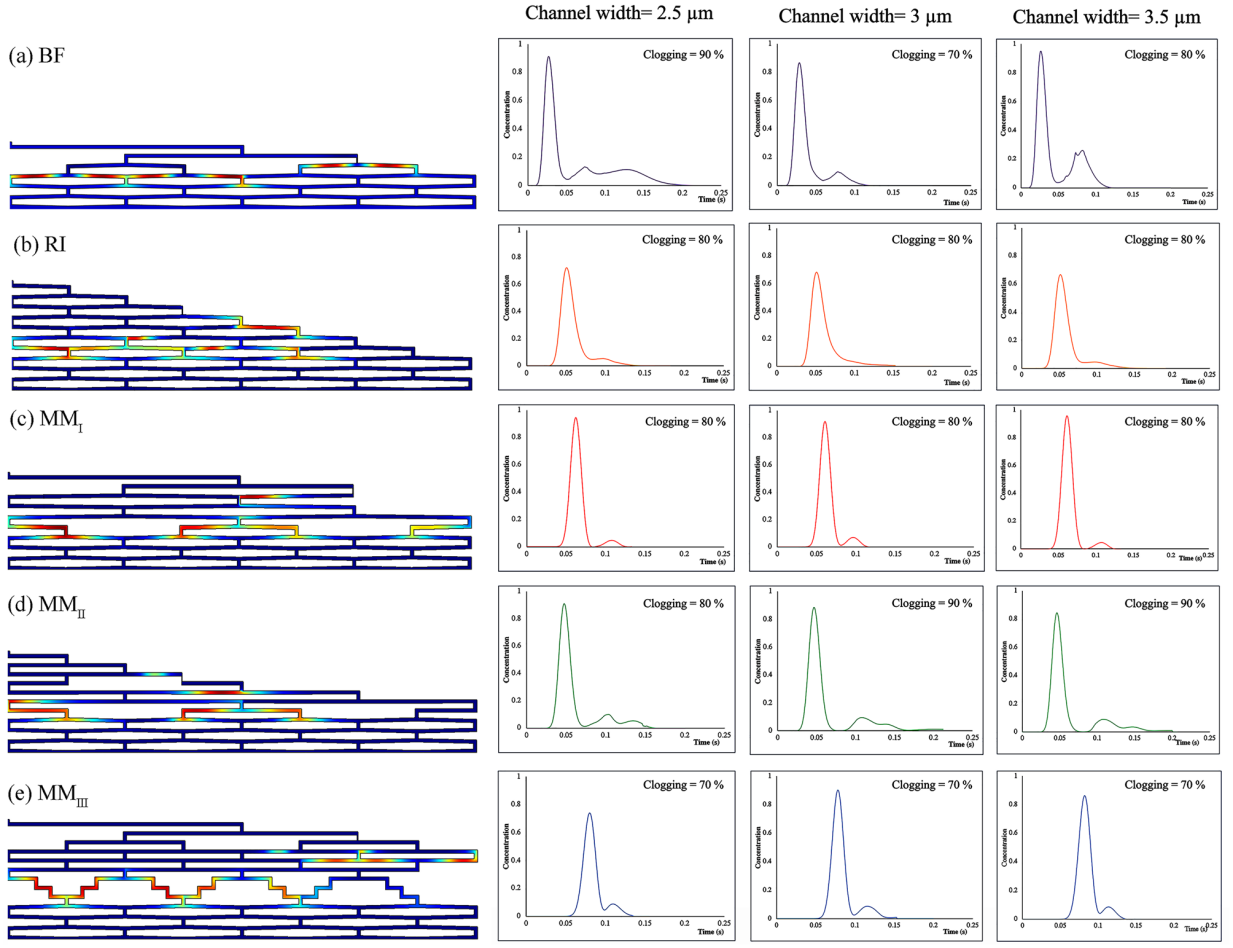
Spatial species distribution prior to the moment of elution in the presence of different degrees of clogging (left). The corresponding chromatograms recorded at the monitor line (right). (AR = 20, flow rate = 1.32 µL/min). Color scales linear with concentration (red = maximum, blue = 0).

Figure 6 shows the concentration profile (channel width = 3 µm) and the corresponding chromatogram of the most affected band because of clogging right before the moment of elution for each flow distributor. The results show that once the clogging occurs, part of the injected sample is trapped in a region near the blockage. Given that the velocity in the clogged channel is much lower than the velocity in other channels, the amount of species that enters the clogged channel takes more time to elute. Therefore, when the degree of clogging exceeds 70%, the eluted peaks either show net tailing with asymmetric behavior or produce extra peaks because of fouling. There is an unexpected drop in σ_v_^2^ value at higher degrees of clogging (Fig. 4) which may be attributed to the small amount of sample entering the blocked region. Due to the lower fluid velocity in the clogged region, it takes more time for the sample to elute which dilute the sample below the detection limit (< 0.1% of the maximum of the peak) by the time it reaches the monitor line. The ripples or waves observed in the BF and MM_II_ chromatograms are similarly interpreted.

Decreasing the channel width increases the Δp value noticeably regardless of the clogging percentage. Therefore, considering the best performance of the flow distributors with the channel width of 3 µm, this channel width was chosen for the rest of the study. Given that the MM_I_ and MM_III_ distributors outperformed under the fouled conditions, the other flow distributors were omitted from further consideration.

### Effect of aspect ratio of the pillars on clogging sensitivity of distributors

**Figure 7:**
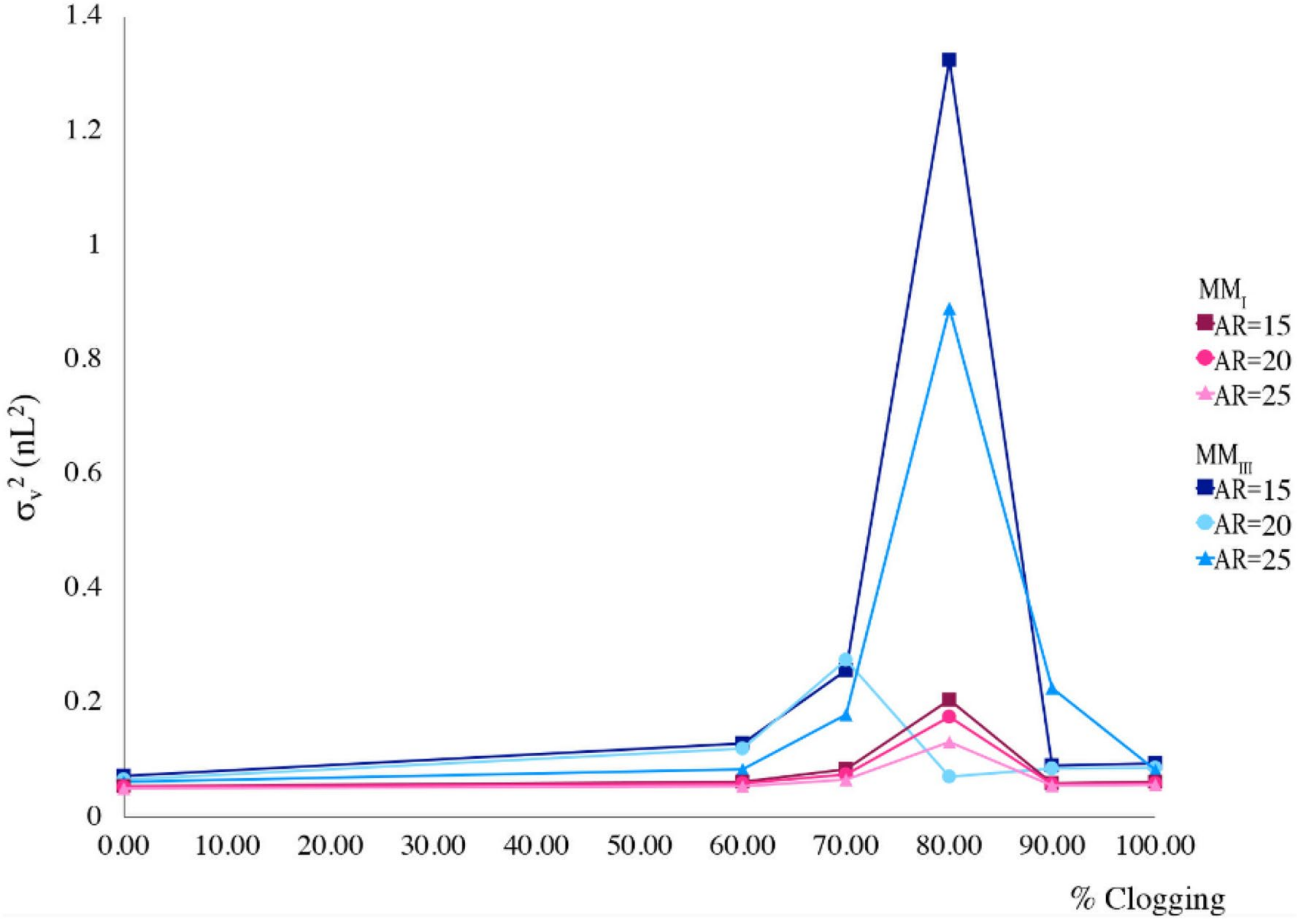
Volumetric variances of the sample band recorded at the monitor line as a function of clogging for MM_I_ and MM_III_ distributors.

The effect of aspect ratio of the pillars on volumetric variances of the tracer peak was evaluated for the MM_I_ and MM_III_ distributors in the presence of clogging (Fig. 7 and Table 3). Three different ARs of 15, 20, 25 were considered. It is obvious that increasing the AR increases the total volume of the distributor. Inlet velocities were adjusted for each flow distributor based on their tortuosity to preserve the flow rate constant at 1.32 µL/min. σ_v_^2^ and m_1_ values were shown to be nearly independent from the considered ARs in the MM_I_ distributor. MM_III_-type, however, produced a broad extra peak after the main peak when the AR was set to 15 or 25. These peaks were eluted below the limit of detection for channel clogging degrees beyond 90%. Additionally, when the degree of clogging increased to 100%, the eluted peaks shifted backwards in the chromatograms, resulted in a drop in the m_1_ values (data not shown).

**Table 3:** Numerical measures comparing MM_I_ and MM_III_ distributors with the channel widths set to 3 µm and with different aspect ratios of the pillars in the presence of clogging at the 4-outlet-level channel. The flow rate is set to 1.32 μL/min in all models. (^a^Mean elution time, ^b^Volumetric variance, ^c^Pressure drop).

Flow distributor	AR	Clogging (%)	m_1_ (s)^a^	${\upsigma }_{\mathrm{v}}^{2}$(nL^2^)^b^	Δp (bar)^c^
MM_I_	15	80	0.064	0.21	0.13
	20	80	0.062	0.17	0.22
	25	80	0.063	0.13	0.32
MM_III_	15	80	0.091	1.33	0.12
	20	70	0.081	0.27	0.19
	25	80	0.087	0.89	0.27

Moreover, the Δp-values over the distributors with different channel widths (same No. of outlets) in the absence and presence of clogging are given in Tables 1 and 3, respectively, indicating that the optimal value for the aspect ratio of the pillars compromise between the pressure drop and volumetric variance. For instance, in MM_I_ designs, increasing the AR decreases σ_v_^2^ value which means the generated peak is sharper, though the pressure drop increases significantly (the same explanation applies to MM_III_ as well). Therefore, MM_I_-type distributor with the channel width of 3 µm and AR = 20 was modified to investigate the effect of the number of outlets (distributor width) and contact zone rows on the ability of the distributor to alleviate the clogging.

Given the temporal change in location of clogging, the influence of several clogged zones induced at the same time were investigated (See Supplementary Information). To this end, two and three channels were modified to induce 80% local clogging simultaneously in MM_1_ distributor. As it was expected, for clogging occurred in more than one area, the volume of entrapped samples became larger, and the extra peak started increasing in intensity (See Fig S1 and S2). Moreover, with the increase in the number of clogged areas, the eluted peaks shifted backwards in the chromatograms, resulted in a drop in the m_1_ values.”

Also, the three-dimensional (3D) MM_I_ distributor with 18 µm channel depth in the absence and presence of 80% degree of clogging at the 4-outlet-level channel was investigated. The concentration profile and the corresponding chromatograms are shown in Figs. S3 and S4, respectively. The results of 3D simulation showed a similar clogging trend behavior as the 2D results, where the entrapped sample near the clogged channel were eluted slower. In real cases, 3D representation of flow distributor, an additional dispersion and flow resistance would be expected due to the top and bottom walls^22,29^. This extra band broadening is proportional to the volume of the distributor and therefore, the time the tracer spends in flow distributor. As a result, the observed differences between distributors in 2D geometry would be enhanced in 3D geometry owing to the contribution of top and bottom wall dispersions in band broadening. Thus, as it was expected, the σ_v_^2^ values of the 3D MM_I_ distributor in the absence (0.10 nL^2^) and presence of 80% clogging (0.33 nL^2^) were twice larger than the values obtained from simulation data of 2D MM_I_ distributor. Although the contribution of the top and bottom walls in band broadening are neglected in the 2D representation of flow distributors, 2D simulation yields an acceptable estimate on clogging trend of each distributor but with much shorter computational run.

### Effect of distributor width and number of contact zone rows on clogging sensitivity

**Figure 8:**
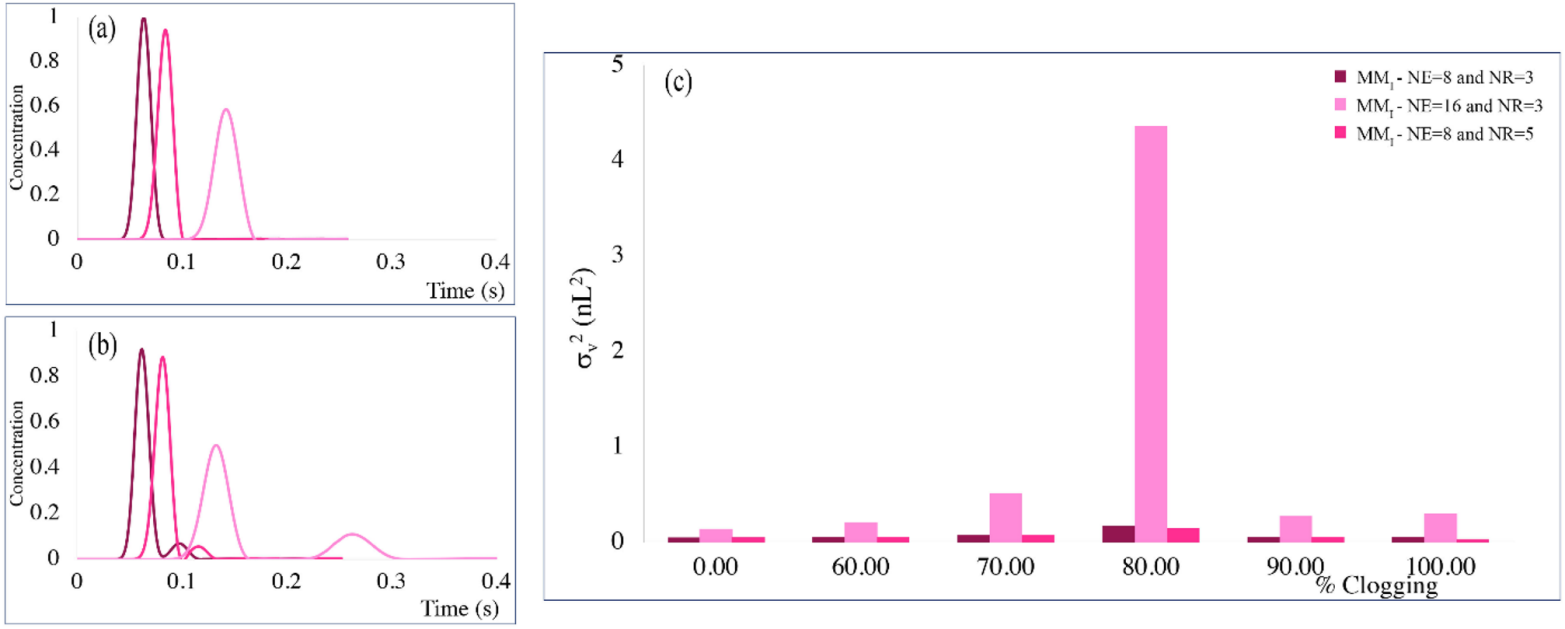
Chromatograms of MM_I_ distributors with different number of outlets and contact zone rows measured at the monitor line (**a**) in the absence of clogging and (**b**) in the presence of 80% clogging of the 4-outlet-level channel. (**c**) volumetric variances of the eluted tracer recorded at the monitor line as a function of the degree of clogging in the red boxes indicated in Fig. 1.

MM_I_-type distributor can only have 2^n^ number of exits (NE) due to its intrinsic structure. MM_I_-type design with channel width of 3 µm and AR = 20 has the total width of 827 µm (NE = 8). Although increasing the NE to 32 leads to a very wide distributor with limited performance (distributor with the width of 1651 µm), the distributor with NE = 16 was investigated to understand the effect of distributor width on compensating the channel fouling. Also, MM_I_-type distributor with NR = 5 was investigated to assess the influence of NR on clogging sensitivity. On the other hand, MM_I_-type distributor has only three rows (NR) of contact zones after the first splitting stage. The liquid velocity was kept constant for all the studied cases and an equal clogging probability was assumed for each distributor. Since the MM_I_ distributor showed inconsiderable difference between the numerical values in the non-clogged and less than 60% clogged conditions, the comparative simulations were performed between the non-clogged states and cases with more than 60% clogging. The results of these simulations are shown in Fig. 8 and the numerical values of the non-clogged cases are summarized in Table 4.

**Table 4:** Numerical measures of non-clogged conditions of the MM_I_ distributor with different numbers of exits and contact zone rows after first splitting stage at the flow rate of 1.32 μL/min. (^a^Mean elution time, ^b^Volumetric variance, ^c^Asymmetry factor, ^d^Tailing factor).

Flow distributor	No. exits (NE)	No. contact zones (NR)	m_1_ (s)^a^	${\upsigma }_{\mathrm{v}}^{2}$(nL^2^)^b^	AsF (/)^c^	TF (/)^d^
MM_I_	8	3	0.064	0.053	1.06	1.06
	16	3	0.142	0.14	0.87	0.99
	8	5	0.083	0.056	0.87	0.91

As the sample volume of the distributor increases through increasing the distributor width or length, the eluted peak becomes wider when the fluid travels further along the distributor toward the monitor line, resulted in a peak broadening and distortion. Therefore, the peak eluted from MM_I_-NE16-NR3-type distributor (σ_v_^2^ = 0.14 nL^2^) is approximately threefold wider than the MM_I_-NE8-NR3 one (σ_v_^2^ = 0.053 nL^2^) (seen in the chromatograms in Fig. 8 (a)) with the longest mean elution time. Furthermore, the peaks eluted from MM_I_-NE8-NR5- and MM_I_-NE16-NR3-type distributors exhibited a strong peak fronting with asymmetry factors about 0.87. Figure 8 (b) shows the resulting chromatograms for 80% clogging of the 4-outlet-level channel in all MM_I_-type distributors. Even though the MM_I_-type distributor exhibits maximum stability and minimum sensitivity towards clogging hitherto, increasing the distributor width (NE = 16) leads to an extra peak or peak distortion after 60% clogging. Unexpectedly, adding to the number of contact zone rows after the first bifurcating stage to overcome the clogging had no significant influence on peak shapes or volumetric variances.

## Discussions and conclusion

One source of “extra-bed” band broadening in micromachined pillar array column is the analyte dispersion by flow distributors^30^. To decrease the band broadening contributed to flow distributor as little as possible, flow distributor needs to be carefully designed and evaluated^31^. Typically, a combination of small sample volume and high radial permeability flow path for the sample band to reach the actual separation channels (column) is the key to achieve the desired uniform distribution^23^. However, another issue that could affect the performance of flow distributor is the local clogging of the microfabricated channels. Therefore, the ideal flow distributor should provide a minimal sample volume and high radial permeability as well as minimal sensitivity to clogging. Given that the flow distributor is located at the entrance of the actual separation bed, the designed flow distributor needs to be integrated into the final structure of the separation bed geometry.

In this work, we employed arrays of radially elongated hexagonal pillars as the separation channels and integrated them with different flow distributor designs to investigate the performance of distributors in alleviating possible local clogging. The selected distributors include BF, RI, and MMs. To characterize the performance of flow distributors in response to channel clogging, employing the CFD model, their ability to distribute the sample band equally throughout the column were studied in the absence and presence of clogging. Since the chance of clogging is higher at the entrance of the microfabricated channels, and these clogged channels affect the performance of the system the most, the 4-outlet-level channel in each distributor was modified to reflect different clogging percentages. Furthermore, the effect of main geometrical design parameters (e.g. channel width, aspect ratio of the pillars, distributor width, and number of contact zone rows) on stability of the distributors in response to different clogging percentages were characterized. It is worth mentioning that in the presence of stationary phase, the analytes are assumed to be diffusing with a rate determined by the stationary phase diffusion coefficient in parallel with that in the mobile phase. Although this phenomenon increases the mean elution time, the trend in clogging would not change. Moreover, the results of this study can be translated to other fluidic systems dealing with clogging challenges, for example in reactors, diagnostics assays, organ-on-chip platforms, and high-throughput drug testing systems.

First, with the number of exits, contact zone rows, and aspect ratio of the pillars fixed, the channel width throughout the geometry varied within 2.5–3.5 µm. According to the Taylor-Aris theory in open-tubular channels, the volumetric variance associated with the dispersion can be expected to be proportional to the 3rd power of the channel width^25^. Since the channel width markedly influences the axial dispersion and the microfabrication of larger channel widths is easier, it is informative to investigate the effect of channel width on the performance of distributors. While varying the channel width has no significant difference in numerical measures of each set of distributors (for example in BF-types) in non-clogged states other than for pressure drop, the behaviour of flow distributors was different in the presence of clogging with varying the channel widths. Because of their structures, BF distributors have the minimum sample volume, therefore they produce the sharpest peaks in the absence of clogging (${\upsigma }_{\mathrm{v}}^{2}\cong 0.046$ nL^2^) with minimum elution times. Nevertheless, with decreasing the dimension of flow paths via decreasing the channel width from 3.5 to 2.5 µm, and increasing the degree of clogging up to 90%, BF with 2.5 µm channel width shows the widest peak (σ_v_^2^ = 2.72 nL^2^). The interconnected flow paths of RI distributors lead to minimum pressure drops ($\ge 0.13$ bar) and sensitivity to different degrees of clogging in the absence or presence of clogging. A lower velocity near the sidewalls of RI distributors, however, results in asymmetric peaks with net tailing. MM distributors show an intermediate behavior in the absence of clogging. The reason of this is that they designed to be the best representatives of BF and RI distributors with minimal volume and ability to alleviate local clogging. In MM_I_ distributor, the rows of the elongated pillars with higher aspect ratio are present in the geometry, giving the fluid more transversal path to distribute. This is the reason of higher pressure drop in MM_I_ distributor. Under changing the channel width in the presence of 80% clogging, MM_II_ distributors are the most affected ones, with more than 12 times higher σ_v_^2^ values than the values in the absence of clogging. The reason for this is that the contact zones after the clogged channel are small which gives the fluid limited time to overcome band distortion. The minimum fluctuations in σ_v_^2^ values occurs as a function of clogging percentages in MM_I_ and MM_III_ distributors owing to the presence of several contact zone rows after the clogged section, giving the distributors the ability to compensate the errors. Accordingly, MM_I_ and MM_III_ distributors with the channel width of 3 µm was selected to study the effect of aspect ratio of pillars on the performance of distributors. It is worth mentioning that no significant changes in the numerical measures at the first four clogging degrees (10, 20, 30, and 40%) was found for all distributors. However, when the degree of clogging exceeds, extra peaks or ripples generated after the main peak due to the lower velocity in the clogged channel. This gives the amount of species that enters the clogged channel more time to elute.

As stated above, one way to enhance the performance of a flow distributor is to increase the radial permeability. This can be achieved through increasing the aspect ratio of the pillars. With the other geometrical parameters fixed, the aspect ratio of the pillars in the separation bed varied within 15–25. When the pillars elongate radially, the length of fluid paths and thus the volume of the distributors increase. This explains why the pressure drop is higher in systems with AR = 25. Furthermore, when the degree of clogging increases to 80%, MM_I_ and MM_III_ distributors produce an extra peak after the main peak, which increases the volumetric variances. This extra peak was broader when the AR was set to 15 or 25 in MM_III_ distributor. Therefore, MM_I_ distributors show the highest ability to compensate the clogging. AR = 20 was chosen given its most efficiency along with suitable pressure drop. Moreover, increasing the MM_I_ distributor’s width or adding the number of contact zone rows may enhance the performance of a distributor in terms of the ability to alleviate the clogging. The increase in the total volume of the distributor, however, increased the volumetric variances, and showed no better performance.

Overall, the MM_I_ distributor with the channel width, the aspect ratio of the pillars, the number of exits, and the number of contact zone rows set to 3, 20, 3, and 8 µm, respectively, demonstrated the highest performance when the clogging occurs.

## Supplementary Information

Supplementary Information
