# Supplementary material for: Clogging sensitivity of flow distributors designed for radially elongated hexagonal pillar array columns: a computational modelling
Source: Sci Rep. 2021 Mar 2;11:4927. doi: 10.1038/s41598-021-84178-w (PMC7925673; doi:10.1038/s41598-021-84178-w)
Supplement: Supplementary file 1 — Supplementary Information [file 41598_2021_84178_MOESM1_ESM.docx]

**Clogging Sensitivity of Flow Distributors Designed for Radially** **Elongated Hexagonal Pillar Array Columns: A Computational Modelling**

**Farideh Haghighi,^1,2^ Zahra Talebpour,^1,^* Amir Sanati-Nezhad^2,^***

^1^ Department of Chemistry, Faculty of Physics and Chemistry, Alzahra University, Vanak, Tehran, Iran

^2^ BioMEMS and Bioinspired Microfluidic Laboratory, Department of Mechanical and Manufacturing, Engineering, Centre for Bioengineering Research and Education (CBRE), Biomedical Engineering Program, University of Calgary, Mechanical Engineering Building, MEB214, 2500 University Dr., N.W., Calgary, AB T2N 1N4, Canada

* [Ztalebpour@alzahra.ac.ir](mailto:Ztalebpour@alzahra.ac.ir)

* [Amir.sanatinezhad@ucalgary.ca](mailto:Amir.sanatinezhad@ucalgary.ca)

**Supplementary Information**


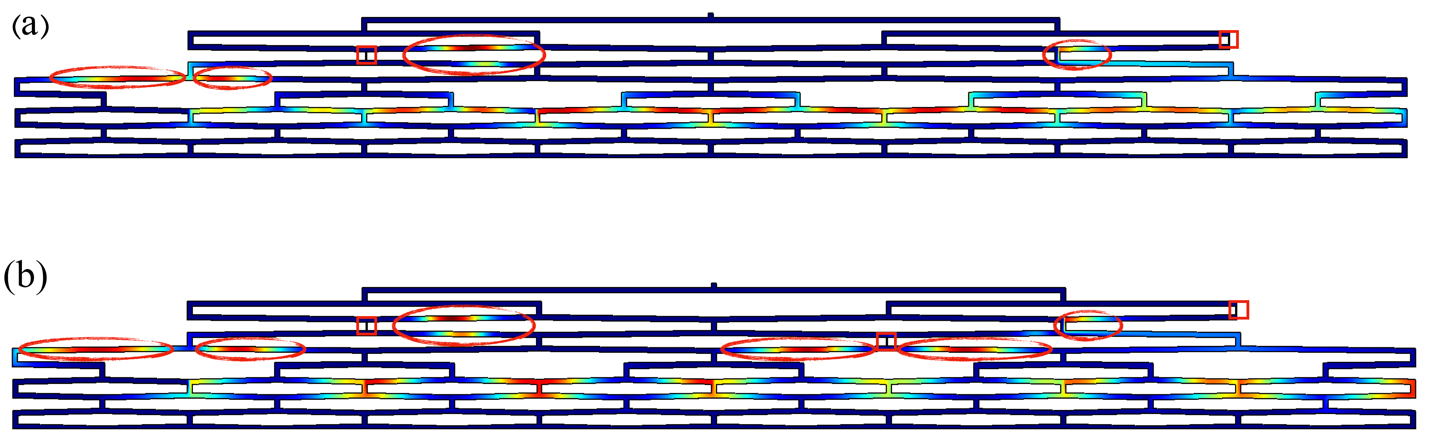


**Fig. S1**. Spatial species distribution prior to the moment of elution in the presence of a) two local clogging and b) three local clogging, simultaneously in MM_I_ distributors. The red boxes exhibit the location of clogging. Color scales linear with concentration (red = maximum, blue = 0).


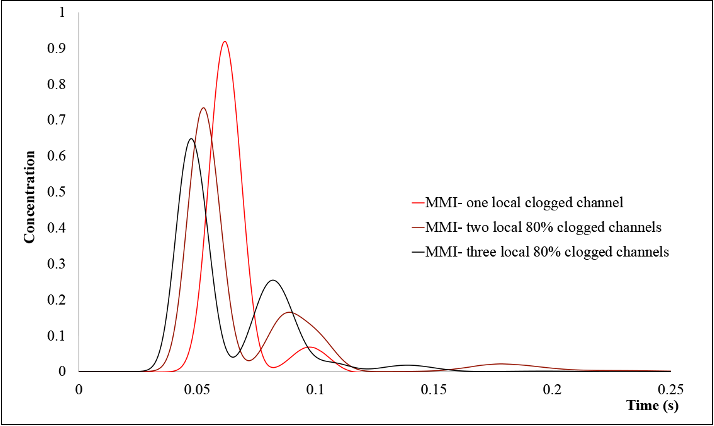


**Fig. S2**. The corresponding chromatograms of MM_I_ distributor recorded at the monitor line in presence of different numbers of clogged channels (Channel width= 3 µm, AR= 20, flow rate= 1.32 µL/min).


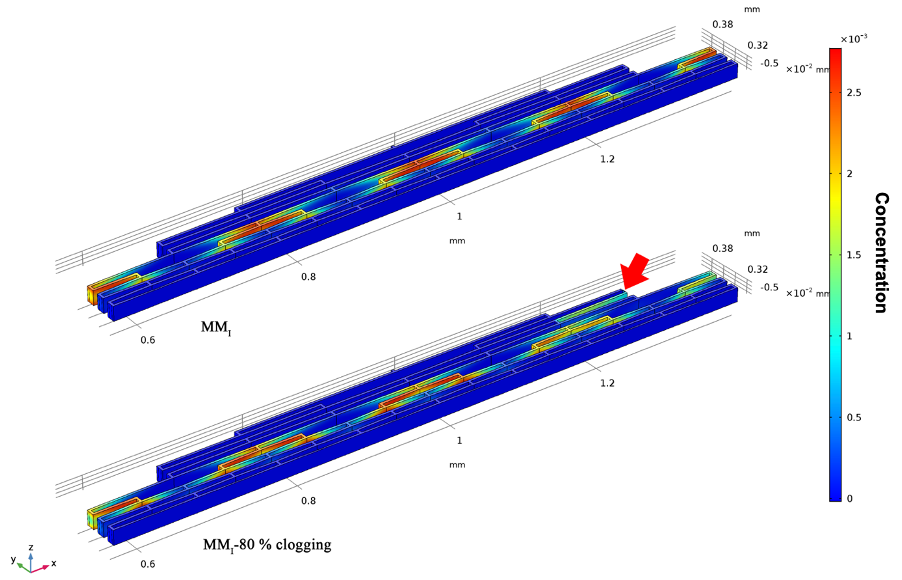


**Fig. S3**. Three-dimensional species distribution prior to the moment of elution in the MM_I_ distributor in the a) absence and b) presence of 80% degree of clogging. The red arrow exhibits the location of clogging. Color scales linear with concentration (red = maximum, blue = 0).


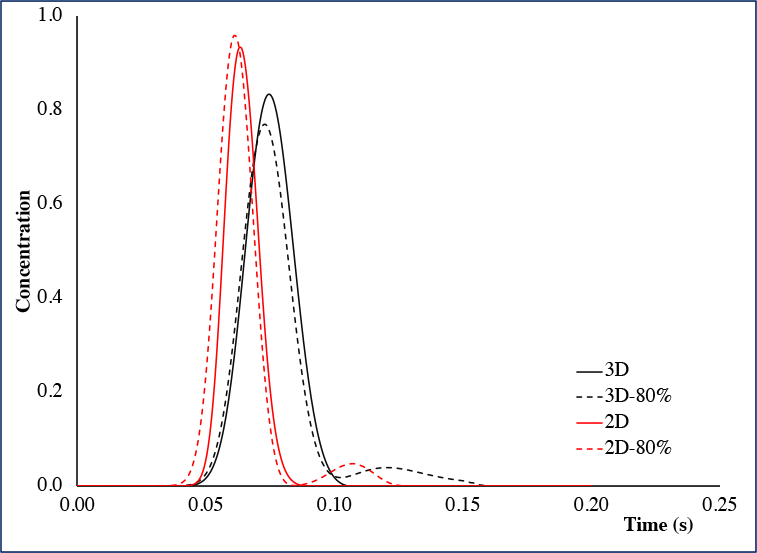


**Fig. S4**. The corresponding chromatograms of two and three-dimensional MM_I_ distributor recorded at the monitor line and plane at the exit of flow distributor in the absence and presence of 80% degree of clogging (Channel width= 3 µm, AR= 20, flow rate= 1.32 µL/min).
